# The Significance of Chloroplast NAD(P)H Dehydrogenase Complex and Its Dependent Cyclic Electron Transport in Photosynthesis

**DOI:** 10.3389/fpls.2021.661863

**Published:** 2021-04-23

**Authors:** Mingzhu Ma, Yifei Liu, Chunming Bai, Jean Wan Hong Yong

**Affiliations:** ^1^College of Land and Environment, National Key Engineering Laboratory for Efficient Utilization of Soil and Fertilizer Resources, Northeast China Plant Nutrition and Fertilization Scientific Observation and Research Center for Ministry of Agriculture and Rural Affairs, Key Laboratory of Protected Horticulture of Education Ministry and Liaoning Province, Shenyang Agricultural University, Shenyang, China; ^2^The UWA Institute of Agriculture, The University of Western Australia, Perth, WA, Australia; ^3^School of Biological Sciences, The University of Western Australia, Perth, WA, Australia; ^4^School of Agriculture and Environment, The University of Western Australia, Perth, WA, Australia; ^5^Liaoning Academy of Agricultural Sciences, Shenyang, China; ^6^Department of Biosystems and Technology, Swedish University of Agricultural Sciences, Alnarp, Sweden

**Keywords:** chloroplast, NAD(P)H, dehydrogenase complex, photosynthesis, cyclic electron transport

## Abstract

Chloroplast NAD(P)H dehydrogenase (NDH) complex, a multiple-subunit complex in the thylakoid membranes mediating cyclic electron transport, is one of the most important alternative electron transport pathways. It was identified to be essential for plant growth and development during stress periods in recent years. The NDH-mediated cyclic electron transport can restore the over-reduction in stroma, maintaining the balance of the redox system in the electron transfer chain and providing the extra ATP needed for the other biochemical reactions. In this review, we discuss the research history and the subunit composition of NDH. Specifically, the formation and significance of NDH-mediated cyclic electron transport are discussed from the perspective of plant evolution and physiological functionality of NDH facilitating plants’ adaptation to environmental stress. A better understanding of the NDH-mediated cyclic electron transport during photosynthesis may offer new approaches to improving crop yield.

## Introduction

Regulation of photosynthetic electron transport in the thylakoid membrane of chloroplasts is fundamental for the maximum photosynthetic yield and plant growth. The light reactions in photosynthesis convert light energy into chemical energy in the forms of ATP and NADPH. The reactions involve two types of electron transport in the thylakoid membrane. While linear electron transport generates both ATP and NADPH, cyclic electron transport around photosystem I (PSI) is exclusively involved in ATP synthesis without the accumulation of NADPH (Johnson, 2011; Yamori et al., 2015). The cyclic electron transport (CET) around PSI includes two distinct and partially redundant pathways in plant chloroplasts. One, i.e., antimycin A-insensitive pathway, is mediated by chloroplast NADH dehydrogenase (NDH) complex (Peltier et al., 2016; Shikanai and Yamamoto, 2017). The other is mediated by PROTON GRADIENT REGULATION5 (PGR5) and PGR5-like Photosynthetic Phenotype1 (PGRL1) protein complex which is sensitive to antimycin A (Munekage et al., 2002, 2004; Dalcorso et al., 2008). Some results have shown that NDH-dependent CET is also involved in plant response to various environmental stresses, such as drought (Munné-Bosch et al., 2005), high temperature (Wang et al., 2006), low temperature (Li et al., 2004; Leonid et al., 2011; Yamori et al., 2011; Wang et al., 2020), low light (Ishikawa et al., 2016a), and phosphorus deficiency (Carstensen et al., 2018; Shi et al., 2019). This review examines the background underlying the research of NDH. The significance of the NDH-mediated cyclic electron transport is discussed from the perspective of plant evolution and physiological functionality of NDH to understand how plants adapt to environmental stress by fine tuning their NDH-mediated cyclic electron transport.

## Discovery of the NDH Complex

Arnon et al. (1954) discovered CET in spinach chloroplasts *in vitro*, but this did not accelerate the understanding of the NDH complex. It was not until 1986 that scientists discovered the NDH complex in the chloroplast genome sequencing of tobacco and liverwort (*Marchantia polymorpha*) (Ohyama et al., 1986; Shinozaki et al., 1986). There were 11 genes (*NdhA*∼*NdhK*) in their chloroplast genome that were highly homologous to the genes encoding the human mitochondrial respiratory chain NADH dehydrogenase complex (Matsubayashi et al., 1987). With these genomic similarities, the NADH dehydrogenase complex in chloroplasts was aptly named as the NAD(P)H dehydrogenase-like complex or commonly referred to as the NDH complex (Yamamoto et al., 2011).

### Structure of the NDH-1 Complex in Cyanobacteria

**FIGURE 1:**
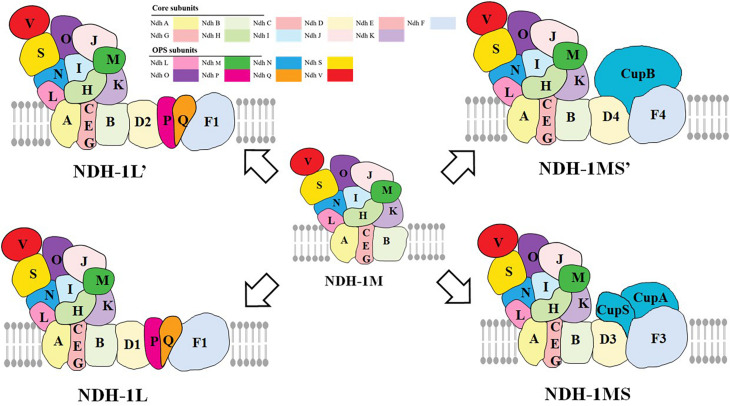
The functional and structural multiplicity of the Cyanobacteria NDH-1 complexes. NDH-1L and NDH-1L′ are involved in respiration and cyclic electron transport around PSI, while NDH-1MS and NDH-1MS′ are involved in the absorption of CO_2_ and cyclic electron transport around PSI, in which NDH-1MS is a low CO_2_-induced CO_2_ absorption complex and NDH-1MS’ is a constitutive CO_2_ absorption complex (adapted from Battchikova et al., 2011; Laughlin et al., 2020; Zhang et al., 2020).

The type I NADH dehydrogenase (NDH-1) is a multisubunit complex located in the thylakoid membrane (Ohkawa et al., 2000), which is widely found in bacteria, cyanobacteria, higher plants, and animals (Friedrich et al., 1995; Yagi et al., 1998; Friedrich and Scheide, 2000; Brandt et al., 2003; Miller et al., 2021). Previous studies have reported that NDH-2 exists widely in bacteria, some in fungi, plants, and protozoa (protist), but it is not involved in respiration and photosynthetic electron transport (Howitt et al., 1999). There are about 26 NDH subunits in cyanobacteria (Laughlin et al., 2020). Proteomic methods and cryoelectron microscopic (cryo-EM) have been used to study the different types of NDH-1 complexes in cyanobacteria, including NDH-1L, NDH1L’, NDH-1MS, and NDH-1MS’ (Figure 1; Peltier et al., 2016; Zhang et al., 2020). The NDH-1L and NDH-1L’ are involved in respiration and the cyclic electron transfer around PSI. In addition, the NDH-1MS and NDH-1MS’ are involved in the absorption of CO_2_ and the cyclic electron transfer around PSI (Ogawa, 1991; Ohkawa et al., 2000). In addition to the NDH-1M component, NDH-1L has two specific subunits NdhD1 and NdhF1. Nowaczyk et al. (2011) found two new subunits: NdhP and NdhQ of NDH-1L in thermophilic cyanobacteria by mass spectrometry, which are located on the membrane arm and play a major role in the stability of NDH-1L (Wulfhorst et al., 2014; Zhao et al., 2015), in which NdhP subunits are unique to NDH-1L (Wulfhorst et al., 2014). The small molecular hydrophilic subunit NdhS and NdhV subunit which can stabilize the binding of NdhS to ferredoxin (Fd) were found in *Synechocystis* sp. (Yamamoto et al., 2011; Zhao et al., 2014). Recently, some researchers reported the cryo-EM structure of the entire NDH-1L complex with all 19 subunits (including NdhV, a transiently associated subunit of NDH-1) and revealed the structure and arrangement of the principal oxygenic photosynthesis-specific (OPS) subunits in the NDH complex (Laughlin et al., 2019; Zhang et al., 2020). The NDH-1L’ complex contains NdhD2 subunit but not NdhD1. The expression level of the complex generally increases under the condition of carbon deficiency (Wang et al., 2004). The subunits of NDH-1MS are CupA, CupS, NdhF3, and NdhD3 (Ohkawa et al., 1998), and the complex has a high affinity for CO_2_, while the specific subunits of NDH-1MS’ are NdhF4, NdhD4, and CupB (Wulfhorst et al., 2014).

### Structure of the Chloroplast NDH Complex

**FIGURE 2:**
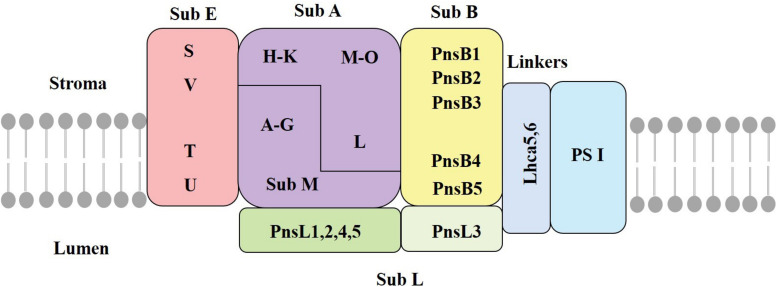
The structure of the chloroplast NDH complex. SubA, subcomplex A; SubB, subcomplex B; SubM, membrane subcomplex; SubL, lumen subcomplex; SubE, electron donor-binding subcomplex; Stroma, thylakoid matrix; Lumen, thylakoid cavity; Lhca5/6, light harvesting pigment protein (adapted from Ifuku et al., 2011; Shikanai, 2016).

The chloroplast NDH complex, located in the thylakoid membrane, mediates CET and chloroplastic respiration (Laughlin et al., 2020). A recent work lists 35 subunits as the presently identified NDH subunits in chloroplasts, of which many have an unknown function. The chloroplast NDH complex is a large thylakoid protein complex composed of 11 chloroplast-encoded subunits (Ndh A∼K) and another 24 nuclear-encoded subunits (Laughlin et al., 2020). These subunits are distributed in different subcomplexes (Sirpio et al., 2009). Previous studies have shown that the NDH complex consists of subcomplex A (SubA), subcomplex B (SubB), lumen subcomplex (SubL), membrane subcomplex (SubM), and electron donor-binding subcomplex (SubE) (Figure 2; Shikanai, 2016). The formation of this supercomplex helps to maintain the stability of the NDH complex under strong light conditions (Peng and Shikanai, 2011). Three subunits of NdhS, NdhT, and NdhU in SubE have been identified through proteomic analysis of NDH-PSI supercomplex (Yamamoto et al., 2011). Fan et al. (2015) identified NdhV as a new subunit of SubE, which is a thylakoid membrane peripheral protein located on the side of the stroma. SubE binds to SubA to form a ferredoxin-binding site; the key function is to bind ferredoxin and facilitating catalysis (Yamamoto et al., 2011).

## NDH-CET in Plant Evolution

### Phycophyta

While examining the NDH-CET from the perspective of plant evolution, we uncovered some salient observations in some phylogenetically primitive organisms. Mi et al. (1992) found inactivated NDH in which the electron transport chain (ETC) from the reduction product produced by the respiratory stroma and the reduction side of PSI to P700^+^ were completely lost in the cyanobacteria mutants. Conversely, in cyanobacterial mutants with partial inactivation of NDH, this ETC was partially inhibited. The CET in cyanobacteria (and not PGR5-CET) occurs mainly *via* the NDH-1 complex (Mi et al., 1995; Yeremenko et al., 2005). Meanwhile, it was found that plant PGR5 suffices to reestablish cyanobacterial cyclic electron transport, albeit less efficiently than the cyanobacterial PGR5 or the plant PGR5 and PGRL1 proteins together (Dann and Leister, 2019; Margulis et al., 2020). Despite the lack of *ndhA*∼*K* gene (encoded by chloroplasts) in *Chlamydomonas reinhardtii*, it still has the ability for CET (Martin et al., 2004). It is plausible that there might be another CET pathways operating in these primitive protists. However, recent data suggest that *Chlamydomonas reinhardtii* performs CET also through the PGR5-PGL1 pathway (Jokel et al., 2018; Yadav et al., 2020). It was proposed that in the process of CET transmission within *Chlamydomonas reinhardtii*, the reduction of plastoquinone (PQ) was facilitated by the NADH: PQ oxidoreductase (a type II NADH: PQ oxidoreductase, Nda2) (Desplats et al., 2009). The structure of Nda2 was considered to be simple, and the catalytic reaction was faster than that of NDH-CET (Kramer and Evans, 2011).

### Plants With C_3_ Mode of Photosynthesis

In *Arabidopsis*, NDH-1 forms a supercomplex with photosystem I and light-harvesting complex I proteins Lhca5 and Lhca6 (Peng et al., 2009; Yadav et al., 2017). Furthermore, the NDH-1-PSI supercomplex has also been identified in barley (Kouřil et al., 2014). Under normal growth conditions, the content of NDH complex in C_3_ plants was lower than that of the thylakoid membrane protein complexes such as PSI, PSII, cytochrome b_6_f (Cytb_6_f), and ATP synthase (Pribil et al., 2014), accounting for only 1.5% of the PSII content (Burrows et al., 1998). The involvement of NDH complex in the CET process was demonstrated using chlorophyll fluorescence parameter kinetics and inhibitor blocking analysis in tobacco *ndh* mutants (Joët et al., 2001). Interestingly, it has been shown that damage of NDH-CET in rice causes a reduction in the electron transport rate through PSI at the low light intensity with a concomitant reduction in CO_2_ assimilation rate (Yamori et al., 2015). Therefore, NDH-CET plays an essential role in normal growth and yield under low light (Rantala et al., 2020).

### Plants With C_4_ Mode of Photosynthesis

During the evolution of plants from C_3_ to C_4_, the expression of NDH increased significantly (Nakamura et al., 2013). The content of NDH in C_4_ plants was higher than that in C_3_ plants, indicating that NDH-CET have a vital role in C_4_ plants (Berger et al., 1993; Ishikawa et al., 2016b). Takabayashi et al. (2005) found that the content of NDH in vascular bundle sheath is 1.6 times higher than that in mesophyll cells of *Scutellaria barbata*. In *Flaveria bidentis*, the content of NDH protein in vascular bundle sheath was three times higher than that in mesophyll cells (Nakamura et al., 2013). In the NADP-ME-type of C_4_ plants, their vascular bundle sheath cells contained more NDH indicating a requirement for more ATP (Friso et al., 2010; Ishikawa et al., 2016b). Darie et al. (2010) not only detected the expression of a new gene (*ndh*E) in maize mesophyll (MS), bundle sheath (BS), and ethioplast (ET) plastids but also found that the NDH complex was divided into 300 kDa subcomplex (corresponding to membrane subcomplex, detected by the NDHE antibody) and 250 kDa subcomplex (detected by the NDHH, -J, and -K antibodies) (Darie et al., 2010). Interestingly, in NAD-ME-type C_4_ plants, the mesophyll cells contained an abundance of NDH protein (Kanai and Edwards, 1999). Besides, chloroplast NDH-1 contains at least 13 additional OPS subunits compared with cyanobacteria, although the current structure of the NDH-1 complex reveals the role of conserved OPS subunits (Laughlin et al., 2019, 2020; Zhang et al., 2020).

## The Physiological Functionality of the NDH Complex

### Providing ATP for Efficient Carbon Assimilation

Theoretically, the NADPH/ATP produced by the linear electron transport is deficient for the assimilation of CO_2_ at different growth stages; the CET pathway, which only produces ATP, but not NADPH, can effectively compensate for this deficiency (Shikanai, 2007; Walker et al., 2016; Nakano et al., 2019). With no NDH activity, the cyanobacteria mutants of *ndhB* (Ogawa, 1990), *ndhH*, *ndhJ*, *ndhN*, and *ndhM* (He et al., 2015), could not survive in normal air CO_2_ concentration; other mutants with partial NDH activity grew slowly in normal air CO_2_ concentration (He and Mi, 2016). The non-functional NDH-CET pathway was attributed to the loss of *ndhB* in plants (Mi et al., 1995). Ogawa (1992) proposed that the NDH-CET pathway provided energy for CO_2_ assimilation and inorganic carbon transport in cyanobacteria. Similarly, the NDH-CET pathway is likely to contribute to the proton motive force (*pmf*) and ATP in chloroplasts of higher plants (Strand et al., 2017). In *Arabidopsis* NDH complex defective mutants, the reduction of *pmf* across the thylakoid membrane led to low availability of ATP. Moreover, the *pmf* produced by NDH-CET was higher than that produced by PGR5/PGRL1-CET (Wang et al., 2015). It was observed that the ATP produced by NDH-CET could effectively compensate for CO_2_ assimilation in a changing environment (Xu et al., 2014; Pan et al., 2020). It was found that the carbon assimilation efficiency of rice mutants (with no NDH activity caused by the lack of CRR6 assembly factor of subcomplex A) is lower than normal rice plants when grown under low light, resulting in a significant decrease in biomass (Yamori et al., 2015). Meanwhile, NDH-CET is also promoted under corresponding stress conditions to adapt to the needs of ATP and ΔpH during changing environment to ensure an effective photosynthetic carbon fixation process (Quiles, 2006).

### Mitigating Oxidative Stress and Stroma Overreduction

It was found that the concentration of NADPH was higher, and more H_2_O_2_ was produced on the acceptor side of PSI, when measuring the NADPH fluorescence kinetics of cyanobacteria NDH mutant (Mi et al., 2000). These observations indicated that NDH-CET plays a key role in the process of antioxidation. Specifically, the NDH-CET initiates photoprotection *via* downregulating electron transport in the Cytb_6_f complex to acidify the thylakoid lumen (Munekage et al., 2004) and induces energy-dependent quenching (qE) component of non-photochemical quenching (NPQ) in PSII to dissipate the absorbed excess light energy. Thus, the oxidative stress of chloroplast can be alleviated and the overreduction of stroma can be prevented (Li et al., 2002). The role of NDH-CET in the process of antioxidation in higher plants principally stems from the study of *ndh* gene knockout in tobacco plastids. Endo et al. (1999) repeatedly irradiated tobacco *ndhB* mutant leaves with strong light and found that the PSII in mutants produced serious photoinhibition. When tobacco leaves were subjected to anaerobic condition, the activity of NDH-CET increased significantly, indicating that it was regulated by the redox state of intersystem electron transporters (Joët et al., 2002). Wang et al. (2006) found that tobacco *ndhC-J-K* mutants accumulated reactive oxygen species more easily than wild types when growing under low- (4°C) or high-temperature (42°C) stress. Chloroplast NDH was able to alleviate oxidative stress in rice under fluctuating light conditions (Yamori et al., 2016), and hydrogen peroxide could be used as a signaling compound to activate NDH-CET (Strand et al., 2015). Moreover, it was shown that the NDH-1-PSI supercomplex consumed electrons for CET as quickly as possible, limiting the space required by Fd_*red*_ diffusion and stabilizing PSI (Gao et al., 2016; Zhao et al., 2018). This accelerated electron consumption is thought to be an antioxidant mechanism, especially when stresses such as high light leads to increased Fd reduction (Miller et al., 2021). These studies suggested that the NDH complex get involved in alleviating the effects of photooxidative stress. Wu et al. (2011) found that a low concentration of NaHSO_3_ promoted NDH-CET in tobacco under a dark-light transition episode, which slowed down the damage of photooxidation while improving plant photosynthesis. These findings revealed that the NDH complex is involved in alleviating the effects of photooxidative stress.

### Regulating the Photosynthetic Apparatus

Generally, PSI is more stable than PSII and less vulnerable to light damage (Sonoike, 2011). *Arabidopsis* (Kono and Terashima, 2016) and rice (Yamori et al., 2016) cannot grow well under fluctuating light due to photoinhibition (Kono et al., 2014); the *pgr5* mutants of *Arabidopsis thaliana* die at the seedling stage under fluctuating light, indicating that the CET pathway has a protective effect on PSI (Suorsa et al., 2016). The relative electron transport rate (ETR) and CO_2_ assimilation rate of rice NDH complex-deleted mutant decreased under long-term low-light and low-temperature conditions (Peng et al., 2009; Peng and Shikanai, 2011; Kono and Terashima, 2016). Conversely, the CET had little regulation on the ratio of ATP/NADPH under high light (Yamori et al., 2015). However, Walker et al. (2014) held an opposite view that CET plays a major role during high light, such as increasing the ATP requirements. In this regard, Huang et al. (2015) deemed that the effect of CET alters in tandem and coinciding with any changes in light intensity. When subjected to subsaturated light intensity, CET is conducive for the formation of proton dynamic potential across the thylakoid membrane, activating ATP synthase to synthesize ATP, while maintaining an optimal ATP/NADPH. Under saturated light, CET provides an important photoprotective role for the activity of oxygen evolution complex (OEC) by forming the proton gradient across the thylakoid membrane (?pH) (Huang et al., 2016b), and *pmf* to protect PSI and PSII *via* the acidification of thylakoid lumen (Golding et al., 2004; Basso et al., 2020). Chilling leads to photoinhibition in cold-sensitive plants like tobacco, peanut, and cucumber which is mainly related to CET activity (Huang et al., 2016a; Liu, 2020; Song et al., 2020; Wu et al., 2020). At 4°C, CET plays a photoprotective role in PSI primarily through the acidification of thylakoid lumen (Huang et al., 2017b). The protective mechanism of CET would alter in accordance with the different growth status of the heliophyte leaves. Under strong light, immature leaves protect the photosystems mainly through the acidification of thylakoid lumen. For the mature leaves, they achieve high light protection through the formation of cross-thylakoid membrane proton gradient, activation of ATP enzyme, and lumen acidification (Huang et al., 2017a). However, Rantala et al. (2020) indicated that PGR5 and NDH-1 systems do not function as protective electron acceptors but mitigate the consequences of PSI inhibition. There is no consensus that the PGR5/PGRL1 compose a true cyclic electron pathway (i.e., acting as electron transporters) mainly due to the lack of solid molecular evidence, although PGR5/PGRL1 seems to be involved in CET at least indirectly (Nawrocki et al., 2019; Rantala et al., 2020).

## Future Outlook

Although the energy provided by NDH-CET is lower than that of LET, it still plays a principal role in fine-tuning energy availability in plants. Besides, it plays a significant role in maintaining photosynthetic carbon fixation of algae and higher plants when encountering abiotic stress events. At present, there are several unanswered questions about NDH-CET: namely, the regulation of NDH pathway which affects the efficient operation of photosynthetic apparatus; the activation of its regulatory mechanism under abiotic stress; the electron transfer processes of NDH; and how they might influence the CO_2_ concentrating mechanism in algae and higher plants. Moving forward, in-depth studies about the NDH-CET pathway are required to improve the light energy utilization efficiency of plants and to further elucidate the mechanism associated with photoprotection. With the availability of newer technology, harnessing these novel and sensitive tools would improve our understanding of the NDH-CET pathway and ultimately help us to improve crop yield and quality.

## Author Contributions

YL, MM, and JY are responsible for the general overview of the opinions stated in the manuscript. YL, CB, and JY wrote and modified the manuscript. All authors reviewed and approved the final version of the submitted manuscript.

## Conflict of Interest

The authors declare that the research was conducted in the absence of any commercial or financial relationships that could be construed as a potential conflict of interest.

## References

[B1] Arnon D. Allen M. Whatley F. R. (1954). Photosynthesis by isolated chloroplasts. *Nature* 174 394–396. 10.1038/174394a0 13194001

[B2] Basso L. Yamori W. Szabò I. Shikanai T. (2020). Collaboration between NDH and KEA3 allows maximally efficient photosynthesis after a long dark adaptation. *Plant Physiol.* 184 2078–2090. 10.1104/pp.20.01069 32978277 PMC7723091

[B3] Battchikova N. Wei L. Du L. Bersanini L. Aro E. M. Ma W. (2011). Identification of novel Ssl0352 protein (NdhS), essential for efficient operation of cyclic electron transport around photosystem I, in NADPH: plastoquinone oxidoreductase (NDH-1) complexes of *Synechocystis* sp. PCC 6803. *J. Biol. Chem*. 286 36992–37001. 10.1074/jbc.M111.263780 21880717 PMC3196108

[B4] Berger S. Ellersiek U. Westhoff P. Klaus S. (1993). Studies on the expression of NDH-H, a subunit of the NAD(P)H-plastoquinone-oxidoreductase of higher-plant chloroplasts. *Planta* 190 25–31. 10.1007/BF00195671

[B5] Brandt U. Kerscher S. Dröse S. Zwicker K. Zickermann V. (2003). Proton pumping by NADH: ubiquinone oxidoreductase. A redox driven conformational change mechanism? *FEBS Lett*. 5451 9–17. 10.1016/S0014-5793(03)00387-9 12788486

[B6] Burrows P. A. Sazanov L. A. Svab Z. Maliga P. Nixon P. J. (1998). Identification of a functional respiratory complex in chloroplasts through analysis of tobacco mutants containing disrupted plastid ndh genes. *EMBO J*. 17 868–876. 10.1093/emboj/17.4.868 9463365 PMC1170436

[B7] Carstensen A. Herdean A. Schmidt S. B. Sharma A. Spetea C. Pribil M. (2018). The impacts of phosphorus deficiency on the photosynthetic electron transport chain. *Plant Physiol.* 177 271–284. 10.1104/pp.17.01624 29540590 PMC5933119

[B8] Dalcorso G. Pesaresi P. Masiero S. Aseeva E. Schünemann D. Finazzi G. (2008). A complex containing PGRL1 and PGR5 is involved in the switch between linear and cyclic electron flow in *Arabidopsis*. *Cell* 132 273–285. 10.1016/j.cell.2007.12.028 18243102

[B9] Dann M. Leister D. (2019). Evidence that cyanobacterial sll1217 functions analogously to pgrl1 in enhancing pgr5-dependent cyclic electron flow. *Nat. Commun*. 10:5299. 10.1038/s41467-019-13223-0 31757966 PMC6876563

[B10] Darie C. C. Biniossek M. L. Winter V. Mutschler B. Haehnel W. (2010). Isolation and structural characterization of the ndh complex from mesophyll and bundle sheath chloroplasts of *Zea mays*. *FEBS J*. 272 2705–2716. 10.1111/j.1742-4658.2005.04685.x 15943805

[B11] Desplats C. Mus F. Cuine S. Billon E. Cournac L. Peltier G. (2009). Characterization of Nda2, a plastoqui-none-reducing type II NAD(P)H dehydrogenase in *Chlamydomonas* chloroplasts. *J. Biol. Chem.* 284 4148–4157. 10.1074/jbc.M804546200 19056727

[B12] Endo T. Shkanai T. Takabayashi A. Asada K. Sato F. (1999). The role of chloroplastic NAD(P)H dehydrogenase in photopro-tection. *FEBS Lett*. 457 5–8. 10.1016/S0014-5793(99)00989-8 10486552

[B13] Fan X. Zhang J. Li W. Peng L. (2015). The NdhV subunit is required to stabilize the chloroplast NADH dehydrogenase-like complex in *Arabidopsis*. *Plant J*. 82 221–231. 10.1111/tpj.12807 25728844

[B14] Friedrich T. Scheide D. (2000). The respiratory complex I of bacteria, archaea and eukarya and its module common with membrane-bound multisubunit hydrogenases. *FEBS Lett.* 479 1–5. 10.1016/S0014-5793(00)01867-6 10940377

[B15] Friedrich T. Steinmüller K. Weiss H. (1995). The proton-pumping respiratory complex I of bacteria and mitochondria and its homologue in chloroplasts. *FEBS Lett.* 367 107–111. 10.1016/0014-5793(95)00548-N 7796904

[B16] Friso G. Majeran W. Huang M. Sun Q. Wijk K. V. (2010). Reconstruction of metabolic pathways, protein expression and homeo-stasis machineries across maize bundle sheath and mesophyll chloroplasts: large-scale quantitative proteomics using the first maize genome assembly. *Plant Physiol.* 152 1219–1250. 10.1104/pp.109.152694 20089766 PMC2832236

[B17] Gao F. Zhao J. Chen L. Battchikova N. Ran Z. Aro E. M. (2016). The NDH-1L-PSI supercomplex is important for efficient cyclic electron transport in cyanobacteria. *Plant Physiol.* 172 1451–1464. 10.1104/pp.16.00585 27621424 PMC5100770

[B18] Golding A. J. Finazzi G. Johnson G. N. (2004). Reduction of the thylakoid electron transport chain by stromal reductants: evidence for activation of cyclic electron transport upon dark adaptation or under drought. *Planta* 220 356–363. 10.1007/s00425-004-1345-z 15316779

[B19] He Z. H. Mi H. (2016). Functional characterization of the subunits N, H, J, and O of the NAD(P)H dehydrogenase complexes in *Synechocystis* sp. strain PCC 6803. *Plant Physiol.* 171 1320–1332. 10.1104/pp.16.00458 27208236 PMC4902626

[B20] He Z. Xu M. Wu Y. Jing L. Mi H. (2015). NdhM is required for the stability and the function of NAD(P)H dehydrogenase complexes involved in CO_2_ uptake in *Synechocystis* sp. strain PCC 6803. *J. Biol. Chem.* 291 5902–5912. 10.1074/jbc.M115.698084 26703473 PMC4786724

[B21] Howitt C. A. Udall P. K. Vermaas W. F. (1999). Type2 NADH dehydrogenases in the cyanobacterium *Synechocystis* sp. strain PCC 6803 are involved in regulation rather than respiration. *J. Bacteriol.* 181 3994–4003. 10.1128/JB.181.13.3994-4003.1999 10383967 PMC93889

[B22] Huang W. Yang Y. J. Zhang S. B. (2017a). Specific roles of cyclic electron flow around photosystem I in photosynthetic regulation in immature and mature leaves. *J. Plant Physiol.* 209 76–83. 10.1016/j.jplph.2016.11.013 28013173

[B23] Huang W. Yang Y. J. Hu H. Zhang S. B. (2015). Different roles of cyclic electron flow around photosystem I under sub-saturating and saturating light intensities in tobacco leaves. *Front. Plant Sci.* 6:923. 10.3389/fpls.015.00923 PMC4621282 26579169

[B24] Huang W. Yang Y. J. Hu H. Zhang S. B. (2016a). Moderate photoinhibition of photosystem II protects photosystem I from photodamage at chilling stress in tobacco leaves. *Front. Plant Sci.* 7:182. 10.3389/fpls.2016.00182 26941755 PMC4761844

[B25] Huang W. Yang Y. J. Hu H. Zhang S. B. Cao K. F. (2016b). Evidence for the role of cyclic electron flow in photoprotection for oxygen-evolving complex. *J. Plant Physiol.* 194 54–60. 10.1016/j.jplph.2016.02.016 26968082

[B26] Huang W. Zhang S. B. Xu C. J. Liu T. (2017b). Plasticity in roles of cyclic electron flow around photosystem I at contrasting temperatures in the chilling-sensitive plant *Calotropis gigantea*. *Environ. Exp. Bot.* 141 145–153. 10.1016/j.envexpbot.2017.07.011

[B27] Ifuku K. Endo T. Shikanai T. Aro E. M. (2011). Structure of the chloroplast NADH dehydrogenase-like complex: nomenclature for nuclear encoded subunits. *Plant Cell Physiol.* 52 1560–1568. 10.1093/pcp/pcr098 21785130

[B28] Ishikawa N. Takabayashi A. Noguchi K. Tazoe Y. Yamamoto H. von Caemmerer S. (2016a). NDH-mediated cyclic electron flow around photosystem I is crucial for C_4_ photosynthesis. *Plant Cell Physiol.* 57 2020–2028. 10.1093/pcp/pcw127 27497446

[B29] Ishikawa N. Takabayashia A. Sato F. Endo T. (2016b). Accumulation of the components of cyclic electron flow around photosystem I in C_4_ plants, with respect to the requirements for ATP. *Photosynth. Res.* 129 261–277. 10.1007/s11120-016-0251-0 27017612

[B30] Joët T. Cournac L. Horvath E. M. Peltier M. G. (2001). Increased sensitivity of photosynthesis to antimycin A induced by inactivation of the chloroplast *ndhB* gene. Evidence for a participation of the NADH-dehydrogenase complex to cyclic electron flow around photosystem I. *Plant Physiol.* 125 1919–1929. 10.1094/PDIS.2000.84.5.594B 11299371 PMC88847

[B31] Joët T. Cournac L. Peltier G. Havaux P. M. (2002). Cyclic electron flow around photosystem I in C_3_ plants. *In vivo* control by the redox state of chloroplasts and involvement of the NADH-dehydrogenase complex. *Plant Physiol.* 128 760–769. 10.2307/4280343 11842179 PMC148937

[B32] Johnson G. N. (2011). Physiology of PSI cyclic electron transport in higher plants. *Biochim. Biophys. Acta* 1807 384–389. 10.1016/j.bbabio.2010.11.009 21118673

[B33] Jokel M. Johnson X. Peltier G. Aro E. M. Allahverdiyeva Y. (2018). Hunting the main player enabling *Chlamydomonas reinhardtii* growth under fluctuating light. *Plant J.* 94 822–835. 10.1111/tpj.13897 29575329

[B34] Kanai R. Edwards G. E. (1999). “The biochemistry of C_4_ photosynthesis,” in *C_4_ Plant Biology*, eds Sage R. F. Monson R. K. (San Diego, CA: Academic Press), 49–87. 10.1016/B978-012614440-6/50004-5

[B35] Kono M. Terashima I. (2016). Elucidation of photoprotective mechanisms of PSI against the fluctuating light photoinhibition. *Plant Cell Physiol.* 57 1405–1414. 10.1093/pcp/pcw103 27354420

[B36] Kono M. Noguchi K. Terashima I. (2014). Roles of the cyclic electron flow around PSI (CEF-PSI) and O_2_-dependent alternative pathways in regulation of the photosynthetic electron flow in short-term fluctuating light in *Arabidopsis thaliana*. *Plant Cell Physiol.* 55 990–1004. 10.1093/pcp/pcu033 24553846

[B37] Kouřil R. Strouhal O. Nosek L. Lenobel R. Chamrád I. Boekema E. J. (2014). Structural characterization of a plant photosystem I and NAD(P)H dehydrogenase supercomplex. *Plant J.* 77 568–576. 10.1111/tpj.12402 24313886

[B38] Kramer D. M. Evans J. R. (2011). The importance of energy balance in improving photosynthetic productivity. *Plant Physiol.* 155 70–78. 10.1104/pp.110.166652 21078862 PMC3075755

[B39] Laughlin T. G. Bayne A. N. Trempe J. F. Savage D. F. Davies K. M. (2019). Structure of the complex I-like molecule NDH of oxygenic photosynthesis. *Nature* 566 411–414. 10.1038/s41586-019-0921-0 30742075

[B40] Laughlin T. G. Savage D. F. Davies K. M. (2020). Recent advances on the structure and function of ndh-1: the complex I of oxygenic photosynthesis. *Biochim. Biophys. Acta Bioenerg.* 1861:148254. 10.1016/j.bbabio.2020.148254 32645407

[B41] Leonid V. S. Alexander G. I. Loreta G. S. Norman P. A. H. John S. (2011). Cold stress effects on PSI photochemistry in *Zea mays*: differential increase of FQR-dependent cyclic electron flow and functional implications. *Plant Cell Physiol.* 52 1042–1054. 10.1093/pcp/pcr056 21546369

[B42] Li X. G. Duan W. Meng Q. W. Zou Q. Zhao S. J. (2004). The function of chloroplastic NAD(P)H dehydrogenase in tobacco during chilling stress under low irradiance. *Plant Cell Physiol.* 45 103–108. 10.1093/pcp/pch011 14749491

[B43] Li X. P. Müller-Moulé P. Gilmore A. M. Niyogi K. K. (2002). Psb S-dependent enhancement of feedback de-excitation protects photosystem II from photoinhibition. *Proc. Natl. Acad. Sci. U.S.A.* 99 15222–15227. 10.1073/pnas.232447699 12417767 PMC137571

[B44] Liu Y. F. (2020). Calcium chemical priming might play a significant role in relieving overnight chilling- dependent inhibition of photosynthesis in crops: a review. *Basic Clin. Pharmacol. Toxicol.* 126 109–110.

[B45] Margulis K. Zer H. Lis H. Schoffman H. Murik O. Shimakawa G. (2020). Over expression of the cyanobacterial Pgr5-homologue leads to pseudoreversion in a gene coding for a putative esterase in synechocystis 6803. *Life* 10:174. 10.3390/life10090174 32899164 PMC7555055

[B46] Martin M. Casano L. M. Zapata J. M. Guéra A. Del Campo E. M. Maier R. M. (2004). Role of thylakoid Ndh complex and peroxidase in the protection against photo-oxidative stress: fluorescence and enzyme activities in wild-type and *ndhF*-deficient tobacco. *Physiol. Plant*. 122 443–452. 10.1111/j.1399-3054.2004.00417.x

[B47] Matsubayashi T. Wakasugi T. Shinozaki K. Yamaguchi-Shinozaki K. Kato A. (1987). Six chloroplast genes (*ndhA-F*) homologous to human mitochondrial genes encoding components of the respiratory chain NADH dehydrogenase are actively expressed: determination of the splice sites in *ndhA* and *ndhB* pre-mR-NAs. *Mol. Gen. Genet.* 210 385–393. 10.1007/BF00327187 3481022

[B48] Mi H. L. Endo T. Asada K. Schreiber U. (1992). Donation of electrons to the intersystem chain in the Cyanobacterium *Synechococcus sp*. PCC 7002 as determined by the reduction of P700^+^. *Plant Cell Physiol.* 33 1099–1105. 10.1093/oxfordjournals.pcp.a078361

[B49] Mi H. L. Endo T. Ogawa T. Asada K. (1995). Thylakoid membrane-bound pyridine nucleotide dehydrogenase complex mediates cyclic electron transport in the cyanobacteria *Synechocystis* PCC 6803. *Plant Cell Physiol.* 36 661–668. 10.1093/oxfordjournals.pcp.a078807

[B50] Mi H. L. Klughammer C. Schreiber U. (2000). Light-induced dynamic of NADPH fluorescence in *Synechocystis* PCC 6803 and its *ndh B*-defective mutant M55. *Plant Cell Physiol.* 41 1129–1135. 10.1093/pcp/pcd038 11148271

[B51] Miller N. T. Vaughn M. D. Burnap R. L. (2021). Electron flow through NDH-1 complexes is the major driver of cyclic electron flow-dependent proton pumping in cyanobacteria. *Biochim. Biophys. Acta Bioenerg.* 1862:148354. 10.1016/j.bbabio.2020.148354 33338488

[B52] Munekage Y. Hashimoto M. Miyake C. Tomizawa K. Endo M. Tasakaet M. (2004). Cyclic electron flow around photosystem I is essential for photosynthesis. *Nature* 429 579–582. 10.1038/nature02598 15175756

[B53] Munekage Y. Hojo M. Meurer J. Endo T. Tasaka M. (2002). PGR5 is involved in cyclic electron flow around photosystem I and is essential for photo-protection in *Arabidopsis*. *Cell* 110 361–371. 10.1016/S0092-8674(02)00867-X 12176323

[B54] Munné-Bosch S. Shikanai T. Asada K. (2005). Enhanced ferredoxin dependent cyclic electron flow around photosystem I and α-tocopherol quinone accumulation in water-stressed ndhB-inactivated tobacco mutants. *Planta* 222 502–511. 10.1007/s00425-005-1548-y 15912357

[B55] Nakamura N. Iwano M. Havaux M. Yokota A. Munekage Y. N. (2013). Promotion of cyclic electron transport around photosystem I during the evolution of NADP-malic enzyme-type C_4_ photosynthesis in the genus *Flaveria*. *New Phytol.* 199 832–842. 10.1111/nph.12296 23627567

[B56] Nakano H. Yamamoto H. Shikanai T. (2019). Contribution of NDH-dependent cyclic electron transport around photosystem I to the generation of proton motive force in the weak mutant allele of pgr5. *Biochim. Biophys. Acta Bioenerg.* 1860 369–374. 10.1016/j.bbabio.2019.03.003 30878346

[B57] Nawrocki W. J. Bailleul B. Cardol P. Rappaport F. Wollman F. A. Joliot P. (2019). Maximal cyclic electron flow rate is independent of pgrl1 in *Chlamydomonas*. *Biochim. Biophys. Acta Bioenerg.* 1860 425–432. 10.1016/j.bbabio.2019.01.004 30711358

[B58] Nowaczyk M. M. Wulfhorst H. Ryan C. M. Souda P. Whitelegge J. P. (2011). NdhP and NdhQ: two novel small subunits of the cyanobacterial NDH-1 complex. *Biochemistry* 50 1121–1124. 10.1021/bi102044b 21244052 PMC3040284

[B59] Ogawa T. (1990). Mutants of *Synechocystis* PCC6803 defective in inorganic carbon transport. *Plant Physiol.* 94 760–765. 10.2307/4273156 16667776 PMC1077296

[B60] Ogawa T. (1991). A gene homologous to the subunit-2 gene of NADH dehydrogenase is essential to inorganic carbon transport of *Synechocystis* PCC6803. *Proc. Natl. Acad. Sci. U.S.A.* 88 4275–4279. 10.1073/pnas.88.10.4275 1903537 PMC51641

[B61] Ogawa T. (1992). Identification and characterization of the *ictA/ndhL* gene product essential to inorganic carbon transport of *Synechocystis* PCC6803. *Plant Physiol.* 99 1604–1608. 10.1104/pp.99.4.1604 16669080 PMC1080670

[B62] Ohkawa H. Pakrkrasi H. B. Ogawa T. (2000). Two types of functionally distinct NAD(P)H dehydrogenases in *Synechocystis* sp strain PCC6803. *J. Biol. Chem.* 275 31630–31634. 10.1074/jbc.M003706200 10906128

[B63] Ohkawa H. Sonoda M. Katoh H. Ogawa T. (1998). The use of mutants in the analysis of the CO_2_-concentrating mechanism in cyanobacteria. *Can. J. Bot.* 76 1035–1042. 10.1139/b98-076

[B64] Ohyama K. Fukuzawa H. Kohchi T. Shirai H. Sano T. Sano S. (1986). Chloroplast gene organization deduced from complete sequence of liverwort *Marchantia polymorpha* chloroplast DNA. *Nature* 322 572–574. 10.1038/322572a0

[B65] Pan X. Cao D. Xie F. Xu F. Li M. (2020). Structural basis for electron transport mechanism of complex i-like photosynthetic NAD(P)H dehydrogenase. *Nat. Commun.* 11:610. 10.1038/s41467-020-14456-0 32001694 PMC6992706

[B66] Peltier G. Aro E. M. Shikanai T. (2016). NDH-1 and NDH-2 plastoquinone reductases in oxygenic photosynthesis. *Annu. Rev. Plant Biol.* 67 55–80. 10.1146/annurev-arplant-043014-114752 26735062

[B67] Peng L. Shikanai T. (2011). Supercomplex formation with photosystem I is required for the stabilization of the chloroplast NADH dehydrogenase-like complex in *Arabidopsis*. *Plant Physiol.* 155 1629–1639. 10.1104/pp.110.171264 21278308 PMC3091109

[B68] Peng L. Fukao Y. Fujiwara M. Takami T. Shikanai T. (2009). Efficient operation of NAD(P)H dehydrogenase requires supercomplex formation with photosystem I via minor LHCI in *Arabidopsis*. *Plant Cell* 21 3623–3640. 10.1105/tpc.109.068791 19903870 PMC2798312

[B69] Pribil M. Labs M. Leister D. (2014). Structure and dynamics of thylakoids in land plants. *J. Exp. Bot.* 65 1955–1972. 10.1093/jxb/eru090 24622954

[B70] Quiles M. J. (2006). Stimulation of chlororespiration by heat and high light intensity in oat plants. *Plant Cell Environ.* 29 1463–1470. 10.1111/j.1365-3040.2006.01510.x 16898010

[B71] Rantala S. Lempiinen T. Gerotto C. Tiwari A. Tikkanen M. (2020). PGR5 and NDH-1 systems do not function as protective electron acceptors but mitigate the consequences of PSI inhibition. *BBA- Bioenergetics*. 1861:148154. 10.1016/j.bbabio.2020.148154 31935360

[B72] Shi Q. Pang J. Yong J. W. H. Bai C. Pereira C. G. Song Q. (2019). Phosphorus-fertilisation has differential effects on leaf growth and photosynthetic capacity of *Arachis hypogaea* L. *Plant Soil.* 447 99–116. 10.1007/s11104-019-04041-w

[B73] Shikanai T. (2007). Cyclic electron transport around photosystem I: genetic approaches. *Annu Rev Plant Biol.* 58 199–217. 10.1146/annurev.arplant.58.091406.110525 17201689

[B74] Shikanai T. (2016). Chloroplast NDH: A different enzyme with a structure similar to that of respiratory NADH dehydrogenase. *Biochim. Biophys. Acta Bioenerg.* 1857 1015–1022. 10.1016/j.bbabio.2015.10.013 26519774

[B75] Shikanai T. Yamamoto H. (2017). Contribution of cyclic and pseudo-cyclic electron transport to the formation of proton motive force in chloroplasts. *Mol. Plant* 10 20–29. 10.1016/j.molp.2016.08.004 27575692

[B76] Shinozaki K. Ohme M. Tanaka M. Wakasugi T. Sugiura M. (1986). The complete nucleotide sequence of the tobacco chloroplast genome: its gene organization and expression. *Plant Mol. Biol. Rep.* 5 2043–2049. 10.1002/j.1460-2075.1986.tb04464.x PMC1167080 16453699

[B77] Sirpio S. Allahverdiyeva Y. Holmstorm M. Khrouchtchova A. Haldrup A. Battchikova N. (2009). Novel nuclear-encoded subunits of the chloroplast NAD(P)H dehydrogenase complex. *J. Biol. Chem.* 284 905–912. 10.1074/jbc.M805404200 18974055

[B78] Song Q. B. Liu Y. F. Pang J. Y. Yong J. W. H. Chen Y. L. Bai C. M. (2020). Supplementary calcium restores peanut (*Arachis hypogaea*) growth and photosynthetic capacity under low nocturnal temperature. *Front. Plant Sci.* 10:1637. 10.3389/fpls.2019.01637 32038667 PMC6985363

[B79] Sonoike K. (2011). Photoinhibition of photosystem I. *Physiol. Plant*. 142 56–64. 10.1111/j.1399-3054.2010.01437.x 21128947

[B80] Strand D. D. Fisher N. Kramer D. M. (2017). The higher plant plastid NAD(P)H dehydrogenase-like complex (n.d.) is a high efficiency proton pump that increases ATP production by cyclic electron flow. *J. Biol. Chem.* 292 11850–11860. 10.1074/jbc.M116.770792 28559282 PMC5512078

[B81] Strand D. D. Livingston A. K. Mio S. C. Froehlich J. E. Kramer D. M. (2015). Activation of cyclic electron flow by hydrogen peroxide *in vivo*. *Proc. Natl. Acad. Sci. U.S.A.* 112 5539–5544. 10.1073/pnas.1418223112 25870290 PMC4418880

[B82] Suorsa M. Rossi F. Tadini L. Labs M. Colombo M. Jahns P. (2016). PGR5-PGRL1-dependent cyclic electron transport modulates linear electron transport rate in *Arabidopsis thaliana*. *Mol. Plant* 9 271–288. 10.1016/j.molp.2015.12.001 26687812

[B83] Takabayashi A. Kishine M. Asada K. Endo T. Sato F. (2005). Differential use of two cyclic electron flows around photosystem I for driving CO_2_ concentration mechanism in C_4_ photosynthesis. *Proc. Natl. Acad. Sci. U.S.A.* 102 16898–16903. 10.1073/pnas.0507095102 16272223 PMC1283823

[B84] Walker B. J. Strand D. D. Kramer D. M. Cousins A. B. (2014). The response of cyclic electron flow around photosystem I to changes in photorespiration and nitrate assimilation. *Plant Physiol.* 165 453–462. 10.1104/pp.114.238238 24664207 PMC4012602

[B85] Walker B. J. Vanloocke A. Bernacchi C. J. Ort D. R. (2016). The costs of photorespiration to food production now and in the future. *Annu. Rev. Plant Biol.* 67 107–129. 10.1146/annurev-arplant-043015-111709 26865340

[B86] Wang C. Yamamoto H. Shikanai T. (2015). Role of cyclic electron transport around photosystem I in regulating proton motive force. *Biochim. Biophys. Acta Bioenerg.* 1847 931–938. 10.1016/j.bbabio.2014.11.013 25481109

[B87] Wang F. Yan J. Wang X. Bu X. Xiang H. Ahammed G. J. (2020). PGR5/PGRL1 and NDH mediate far-red light-induced photoprotection in response to chilling stress in tomato. *Front. Plant Sci.* 11:669. 10.3389/fpls.2020.00669 32547581 PMC7270563

[B88] Wang H. L. Postier B. L. Burnap R. L. (2004). Alterations in global patterns of gene expression in *Synechocystis* sp. PCC 6803 in response to inorganic carbon limitation and the inactivation of ndhR, a LysR family regulator. *J. Biol. Chem.* 279 5739–5751. 10.1074/jbc.M311336200 14612435

[B89] Wang P. Duan W. Takabayashi A. Endo T. Mi H. (2006). Chloroplastic NAD(P)H dehydrogenase in tobacco leaves functions in alleviation of oxidative damage caused by temperature stress. *Plant Physiol.* 141 465–474. 10.1104/pp.105.070490 16428601 PMC1475475

[B90] Wu D. Liu Y. Pang J. Yong J. W. H. Chen Y. Bai C. (2020). Exogenous calcium alleviates nocturnal chilling-induced feedback inhibition of photosynthesis by improving sink demand in peanut (*Arachis hypogaea*). *Front. Plant Sci.* 11:607029. 10.3389/fpls.2020.607029 33408732 PMC7779555

[B91] Wu Y. X. Zheng F. F. Ma W. M. Han Z. G. Gu Q. Shen Y. K. (2011). Regulation of NAD(P)H dehydrogenase-dependent cyclic electron transport around PSI by NaHSO_3_ at low concentrations in tobacco chloroplasts. *Plant Cell Physiol.* 52 1734–1743. 10.1093/pcp/pcr109 21828103

[B92] Wulfhorst H. Franken L. E. Wessinghage T. Boekema E. J. Nowaczyk M. M. (2014). The 5 kDa protein NdhP is essential for stable NDH-1L assembly in *Thermosynechococcus elongatus*. *PLoS One* 9:e103584. 10.1371/journal.pone.0103584 25119998 PMC4131877

[B93] Xu M. Shi N. Li Q. Mi H. (2014). An active supercomplex of NADPH dehydrogenase mediated cyclic electronflow around photosystem I from the panicle chloroplast of *Oryza sativa*. *Acta Biochim. Biophys. Sin.* 46 757–765. 10.1093/abbs/gmu064 25074414

[B94] Yadav K. N. S. Semchonok D. A. Luká N. Kouřil R. Fucile G. Boekema E. J. (2017). Supercomplexes of plant photosystem I with cytochrome b6f, light-harvesting complex II and NDH. *Biochim. Biophys. Acta Bioenerg.* 1858 12–20. 10.1016/j.bbabio.2016.10.006 27755973

[B95] Yadav R. M. Aslam S. M. Madireddi S. K. Chouhan N. Subramanyam R. (2020). Role of cyclic electron transport mutations *pgrl1* and *pgr5* in acclimation process to high light in *Chlamydomonas reinhardtii*. *Photosynth. Res*. 146 247–258. 10.1007/s11120-020-00751-w 32350701

[B96] Yagi T. Yano T. Di Bernardo S. Matsunoyagi A. (1998). Procaryotic complex I (NDH-1), an overview. *Biochim. Biophys. Acta Bioenerg.* 1364 125–133. 10.1016/S0005-2728(98)00023-1 9593856

[B97] Yamamoto H. Peng L. Fukao Y. Shikanai T. (2011). An Src homology 3 domain-like fold protein forms a ferredoxin binding site for the chloroplast NADH dehydrogenase-like complex in *Arabidopsis*. *Plant Cell* 23 1480–1493. 10.1105/tpc.110.080291 21505067 PMC3101538

[B98] Yamori W. Makino A. Shikanai T. (2016). A physiological role of cyclic electron transport around photosystem I in sustaining photosynthesis under fluctuating light in rice. *Sci. Rep.* 6:20147. 10.1038/srep20147 26832990 PMC4735858

[B99] Yamori W. Sakata N. Suzuki Y. Shikanai T. Makino A. (2011). Cyclic electron flow around photosystem I via chloroplast NAD(P)H dehydrogenase (NDH) complex performs a significant physiological role during photosyn-thesis and plant growth at low temperature in rice. *Plant J.* 68 966–976. 10.1111/j.1365-313X.2011.04747.x 21848656

[B100] Yamori W. Shikanai T. Makino A. (2015). Photosystem I cyclic electron flow via chloroplast NADH dehydrogenase-like complex performs a physiological role for photosynthesis at low light. *Sci. Rep.* 5:13908. 10.1038/srep15593 26358849 PMC4566099

[B101] Yeremenko N. Jeanjean R. Prommeenate P. Vladimir K. Nixon P. J. Vermaas W. (2005). Open reading frame ssr2016 is required for antimycin A-sensitive photosystem I-driven cyclic electron flow in the cyanobacterium *Synechocystis* sp. PCC 6803. *Plant Cell Physiol.* 46 1433–1436. 10.1093/pcp/pci147 15946981

[B102] Zhang C. Shuai J. Ran Z. Zhao J. Lei M. (2020). Structural insights into ndh-1 mediated cyclic electron transfer. *Nat. Common.* 11:888. 10.1038/s41467-020-14732-z 32060291 PMC7021789

[B103] Zhao J. Gao F. Fan D. Y. Chow W. S. Ma W. (2018). NDH-1 is important for photosystem I function of *Synechocystis* sp. strain PCC 6803 under environmental stress conditions. *Front. Plant Sci.* 8:2183. 10.3389/fpls.2017.02183 29387069 PMC5776120

[B104] Zhao J. Gao F. Qiu Z. Wang Q. X. Ma W. M. (2014). Deletion of an electron donor-binding subunit of the NDH-1 complex, Ndh S, results in a heat-sensitive growth phenotype in *Synechocystis* sp. PCC 6803. *Chin. Sci. Bull.* 59 4484–4490. 10.1007/s11434-014-0596-8

[B105] Zhao J. Rong W. Gao F. Ogawa T. Ma W. (2015). Subunit Q is required to stabilize the large complex of NADPH dehydrogenase in *Synechocystis* sp. strain PCC 6803. *Plant Physiol.* 168 443–451. 10.1104/pp.15.00503 25873552 PMC4453799

